# Diagnostic potential of a circulating miRNA model associated with therapeutic effect in heart failure

**DOI:** 10.1186/s12967-022-03465-w

**Published:** 2022-06-11

**Authors:** Lu Qian, Qian Zhao, Ping Yu, Jinhui Lü, Yuefan Guo, Xin Gong, Yuanyuan Ding, Shanshan Yu, Lieying Fan, Huimin Fan, Yuzhen Zhang, Zhongmin Liu, Hongzhuan Sheng, Zuoren Yu

**Affiliations:** 1 Key Laboratory of Arrhythmias of the Ministry of Education of China, Research Center for Translational Medicine, Heart Failure Institute, Shanghai East Hospital, Jinzhou Medical University & Tongji University School of Medicine, 150 Jimo Road, Shanghai, 200120 China; 2 Affiliated Hospital of Nantong University, Nantong, Jiangsu China

**Keywords:** Heart failure, Diagnosis, Circulating miRNA, Biomarker

## Abstract

Heart failure (HF), as the leading cause of death, is continuing to increase along with the aging of the general population all over the world. Identification of diagnostic biomarkers for early detection of HF is considered as the most effective way to reduce the risk and mortality. Herein, we collected plasma samples from HF patients (n = 40) before and after medical therapy to determine the change of circulating miRNAs through a quantitative real-time PCR (QRT-PCR)-based miRNA screening analysis. miR-30a-5p and miR-654-5p were identified as the most significantly changed miRNAs in the plasma of patients upon treatment. In consistence, miR-30a-5p showed upregulation and miR-654-5p showed downregulation in the circulation of 30 HF patients, compared to 15 normal controls in the training phase, from which a two-circulating miRNA model was developed for HF diagnosis. Next, we performed the model validation using an independent cohort including 50 HF patients and 30 controls. As high as 98.75% of sensitivity and 95.00% of specificity were achieved. A comparison between the miRNA model and NT-pro BNP in diagnostic accuracy of HF indicated an upward trend of the miRNA model. Moreover, change of the two miRNAs was further verified in association with the therapeutic effect of HF patients, in which miR-30a-5p showed decrease while miR-654-5p showed increase in the plasma of patients after LVAD implantation. In conclusion, the current study not only identified circulating miR-654-5p for the first time as a novel biomarker of HF, but also developed a novel 2-circulating miRNA model with promising potentials for diagnosis and prognosis of HF patients, and in association with therapeutic effects as well.

**Supplementary Information:**

The online version contains supplementary material available at 10.1186/s12967-022-03465-w.

## Introduction

Heart failure (HF), as one of the most common causes of morbidity and mortality worldwide, is the ultimate result of most myocardial and vascular diseases including cardiomyopathies, myocardial infarction (MI), myocarditis, and functional heart disorders derived from hypertension, diabetes, infections or cardio-toxic drugs. Patients with HF suffer from symptoms of insufficient oxygen supply, dyspnea, arrhythmia, fatigue and weakness, edema and fluid retention, and reduced ability to exercise mainly due to impaired left ventricle (LV) myocardial dysfunction [1, 2]. Currently, diagnosis of heart failure mostly relies on the physical examination and lab test, including the concentration of N-terminal pro-B-type natriuretic peptide (NT-proBNP) in blood and ejection fraction (EF) value of the heart [1, 2]. For the patients with HF, a > 30% NT-proBNP reduction after treatment predicts a good prognosis. And ≤ 30% reduction at discharge is always considered as a significant predictor of readmissions and mortality [3]. However, changes of these diagnostic parameters occur only after functional or structural damage of the heart in patients. Identification of novel biomarkers for early prediction of HF is believed to be the most effective way to prevent HF development and/or slow down HF progression in the patients with cardiovascular disorders.

Small non-coding microRNAs (miRNAs), such as miR-1, miR-133, miR-208, and miR-301a have been reported to have important function in regulating heart development, heart-related diseases and LV remodeling [4–6]. Circulating miRNAs in body fluids have been demonstrated to have potential as diagnostic and prognostic biomarkers in diverse diseases including human cancers and cardiovascular diseases [7–11]. For example, a randomized Multicenter Italian Lung Detection (MILD) clinical trial study using 939 participants demonstrated a plasma-based miRNA signature classifier (MSC) has predictive, diagnostic, and prognostic value, and thus improving the efficacy of lung cancer screening [12]. According to the information from the website of Clinicaltrials, a number of clinical studies have been registered for miRNAs as diagnostic or prognostic biomarkers in diverse diseases including coronary heart disease, diabetes, influenza, and multiple types of human cancer [13].

A number of miRNAs have been reported to have potential and utility as biomarkers for predicting HF progression or evaluating the LV function [8–11, 14–19]. A myocardium-enriched miRNA, miR-499 had an increased level in the blood circulation of patients with acute myocardial infarction (AMI) [9, 16, 17]. Moreover, the increased level of miR-499 was present in plasma of patients earlier than other traditional AMI biomarkers like SMB, cTnI, cTnT, CK-MB, CK and LDH, suggesting its potential for early detection of AMI [17]. In addition to miR-499, miR-1, miR-133a/b, and miR-30a were showed increase in the plasma of AMI patients, and in correlation with the cardiac damage degree [8, 9, 18, 20]. Maciejak A. et al. [8] identified circulating miR-30a-5p as a prognostic biomarker of the LV dysfunction after AMI by using a screening analysis and independent validation. miR-30a-5p showed significant increase in the plasma of patients with LV dysfunction and HF symptoms 6 months after AMI [8]. Another analysis by Pergola V. et al. [21] indicated the higher levels of circulating miR-30a and miR-21 in the patients with non-ischaemic HF, while lower levels of circulating miR-423 and miR-34a in the patients with ischaemic HF, suggesting a selective secretion of miRNAs by the damaged heart into the coronary circulation.

In the current study, we performed a miRNA screening analysis using HF inpatients’ plasma samples, and compared the paired samples between the time of check-in before any medical treatment and the time of check-out after partial or complete recovery. A subset of circulating miRNAs was identified to associate with medical treatment. miR-30a-5p and miR-654-5p were subsequently applied to plasma samples from HF patients and normal controls for the independent training and validation analyses. As a result, a novel 2-circulating miRNA model was developed, showing a high sensitivity of 98.75% and high specificity of 95.00% (AUC of 0.9978) for prognosis of HF. Moreover, changes of the two miRNAs were further verified in association with the therapeutic effect of HF patients before and after LVAD implantation.

## Materials and methods

### Phase definition

We applied three phases in this study. Discovery phase refers to the initial screening step of the study. Training phase was applied to confirm the results found in the discovery phase, and used to develop a diagnostic model. Validation phase was a larger independent cohort to further validate the diagnostic model developed in the training phase.

### Patient cohorts

Patients were diagnosed as HF and admitted in hospital at Shanghai East Hospital. All the inpatients received echocardiography analysis and blood lab tests at Shanghai East Hospital. According to the “Guidelines for the diagnosis and treatment of acute and chronic heart failure” [22], only those HF patients with EF ≤ 50% and NT-proBNP ≥ 450 pg/mL if less than 55 years old, or ≥ 900 pg/mL between 55 and 75 years old, or ≥ 1800 pg/mL if over 75 years old, and without other diseases were enrolled in the study.

In the discovery cohort (n = 40), only those patients were included when NT-proBNP decreased at least 30% after medical treatment with partial or complete LVEF recovery when leaving hospital, compared to the NT-proBNP value at check-in. According to the definition for identifying HF patients with a recovered LVEF by the JACC Scientific Expert Panel [23], patients were considered as complete recovery of LVEF when EF > 50% or partial recovery when EF = 40–50%. The 30% NT-proBNP reduction was determined according to the Expert Consensus of Clinical Application of NT-proBNP [24].

Medicines including β-receptor blocker, spironolactone, and sacubitril valsartan sodium tablets were given to those enrolled inpatients under the guidance of specialized doctors at Shanghai East Hospital. In the training cohort, 30 patients and 15 normal controls were enrolled. In the validation cohort, 50 patients and 25 normal controls were enrolled for the diagnostic model validation. Subjects in the training cohort and validation cohort were from the same hospital, but enrolled and organized by different physicians at different time period. Those HF patients with other diseases, such as diabetes and cancer, or having other medical treatment were exclusive from the enrollment of this study. A small RNA sequencing dataset from 27 patients with advanced heart failure without LVAD implantation, 10 patients with advanced heart failure with LVAD implantation for 3 months and 10 patients with advanced heart failure with LVAD implantation for 6 months [15] were applied to further validate the changes of miR-30a-5p and miR-654-5p before and after medical therapy.

Clinical characteristics of the HF patient cohorts in the discovery, training and validation phases were listed in Additional file 1: Tables S1 and S2. The HF diagnosis was performed according to the World Health Organization standard diagnostic procedure. The HF stages were classified according to the symptoms of the patients following the guideline of New York Heart Association (NYHA) Functional Classification. The study was approved by the Institutional Review Board (IRB) of Shanghai East Hospital, Tongji University School of Medicine. All subjects were provided a written informed consent.

### Determination of sample size

Following the principle of diagnostic studies, we calculated the sample size in training phase using the formula $$N(HF)=\frac{{Z\alpha }^{2}*Sn*\left(1-Sn\right)}{{\delta }^{2}}$$ and $$N(control)=\frac{{Z\alpha }^{2}*Sp*\left(1-Sp\right)}{{\delta }^{2}}$$ (Sn: sensitivity; Sp: specificity). As a result, a minimum size for the normal control group of 38 and minimum size for the HF group of 20 were obtained. In our study, 40 normal samples and 80 HF patient samples were applied to develop the diagnostic model.

### Plasma collection and RNA extraction

Blood samples were collected into the EDTA-treated tubes from HF patients and normal controls at Shanghai East Hospital, followed by immediate centrifugation at the speed of 2000 rpm for 5 min at 4 °C. The supernatant plasma was stored in −80 ℃ freezer. Taking an aliquot of 200 µl for total RNA extraction by using 1 mL of Trizol reagent (Invitrogen, USA) following the standard protocol. Glycogen was used as an inert carrier to make RNA pellet visible. The quality of RNA was analyzed using Agilent Bioanalyzer 2100. All the procedures were approved by the Institutional Review Board (IRB) of Shanghai East Hospital, Tongji University School of Medicine.

### miRNA QRT-PCR analysis

200 ng of total plasma RNA was applied to prepare the first strand cDNA of miRNAs by using the M&G miRNA Reverse Transcription kit (miRGenes, China) following the manufacturer’s instruction. The SYBR Green Master Mix (Applied Biosystem, USA) and QuantStudio™ 6 Flex Real-Time PCR System (Applied Biosystem, USA) were used for real-time PCR analysis. 5 s rRNA was used for normalization. Forward primer sequences for miR-30a-5p: 5′uguaaacauccucgacug 3′; miR-654-5p: 5′ugggccgcagaacaugu 3′; 5 s rRNA, 5′ agtacttggatgggagaccg 3′. All primer oligos were synthesized by GenScript (Nanjing, China).

### Diagnostic model development

Binary logistic regression was applied for development of the miRNA diagnostic models by using IBM SPSS Statistics 26 software. The relationship between dependent Y scores (Control and HF as variables) and independent values (miR-30a-5p and miR-654-5p as variables) was analyzed. Based on relevant parameters in the binary logistic regression, three mathematical diagnostic models were developed, in which Y score 0.5 was set as cutoff. The samples were judged as HF if Y scores greater than 0.5, otherwise as normal.

### miRNA target gene prediction and pathway analysis

The ENCORI tool (https://starbase.sysu.edu.cn/index.php) was used to predict the target genes of miR-30a-5p and miR-654-5p. Pathway analyses were performed using web-based gene set analysis toolkit (WebGestalt, http://www.webgestalt.org/).

### Statistical analysis

Two-tailed p-values were calculated using paired samples t-tests in the discovery phase. Two-tailed t-test was used to analyzing the independent samples in the training and validation phases. The public datasets GSE53080 and GSE52601 were obtained from the NCBI-GEO database, in which the read counts were converted to transcripts per million (TPM) by using Python 3.7.4 software. For statistical analysis of the miRNA expression, log transformation of the values (2^-∆∆Ct) was applied in order to obtaining normal distribution of the miRNA expression levels [25, 26]. Receiver operating characteristic (ROC) curves were drawn to calculate the area under the curve (AUC) and assess the diagnostic values using GraphPad Prism V8.0 software. p < 0.05 was considered as statistically significant difference.

## Results

### Characteristics of subjects

**Fig. 1:**
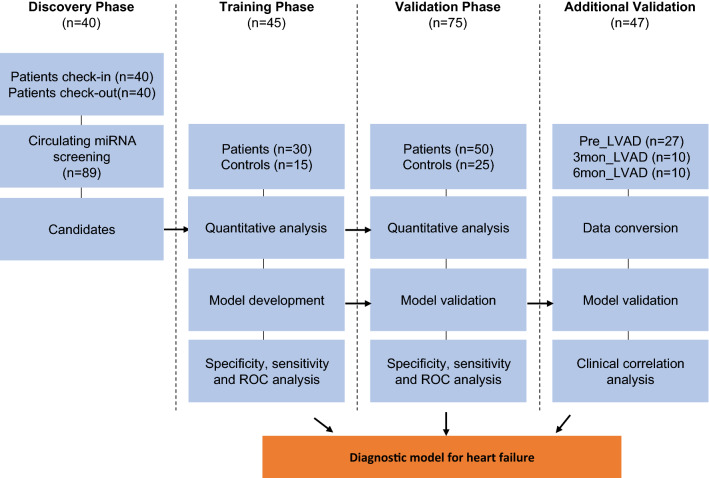
Overview of the study design

A total of 160 subjects, including 120 HF patients and age-matched 40 normal controls were included in the current study to determine the circulating miRNA signature for diagnosis of HF patients. All the characteristics of the patients in the discovery phase, training phase and validation phase were shown in Additional file 1: Table S1 and S2. The flow chart of the study design was shown in Fig. 1.

### Identification of the medical therapy-associated circulating miRNAs in HF patients

**Fig. 2:**
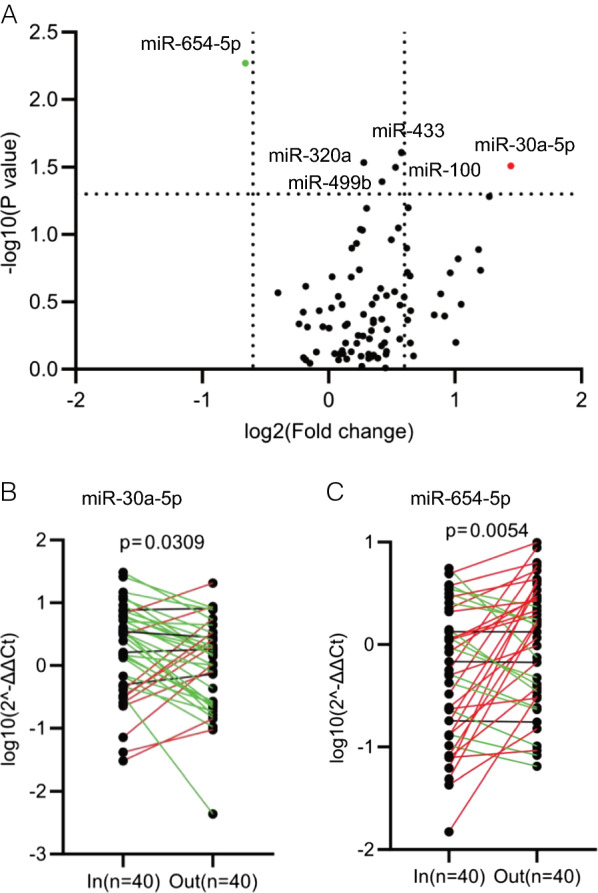
Identification of the medical therapy-associated circulating miRNAs in heart failure (HF) patients. **A** QRT-PCR based screening of circulating miRNAs in the plasma samples of HF patients (n = 40) before and after medical treatment. Comparisons were made by before treatment vs after treatment. Six miRNAs were identified to be significantly associated with the therapeutic effect, including miR-654-5p with upregulation and miR-30a-5p, 449b, 320a, 433 and 100 with downregulation after treatment. By applying cutoffs with fold change (FC) > 1.5 and p < 0.05, miR-30a-5p and miR-654-5p were screened out as the most significantly changed miRNAs. **B**, **C** Expression patterns of miR-30a-5p (**B**) and miR-654-5p (**C**) in each inpatient in the discovery cohort with paired plasma samples collected at times of check-in and check-out. Two-tailed p-values were calculated using paired samples t-tests

In order to identify the circulating miRNAs associated with HF progression and medical treatment, 40 HF inpatients were selected for plasma collection and circulating miRNAs screening analysis. All of these patients had NT-proBNP decreased at least 30% after therapy when leaving hospital, compared to the values at check-in. Comparative analyses of the circulating miRNA levels before and after medical treatment were performed for all the 40 patients. A QRT-PCR-based home-made miRNA panel containing 89 cardiovascular-related human miRNAs (Additional file 1: Table S3) were applied. As a result, a subset of six miRNAs were found to have significant change (p < 0.05) in abundance in the plasmas (Fig. 2A, before treatment *vs* after treatment). Among them, miR-30a-5p, miR-100, miR-499b, miR-320a and miR-433, showed significant downregulation, while miR-654-5p showed upregulation in plasma of those patients after therapy. After applying cutoffs of absolute fold change (|FC|) > 1.5 and p-value < 0.05, miR-30a-5p and miR-654-5p were screened out as the most significantly changed candidate miRNAs of our interest (Fig. 2A). The expression patterns of the six circulating miRNAs in the paired plasma samples indicated higher levels of miR-30a-5p, -100, -499b, -320a and -433 and lower level of circulating miR-654-5p in the HF patients before therapy, while changed to an opposite direction after medical therapy (Additional file 1: Figure S1, Fig. 2B and C). The expression patterns and change trends of the rest of the circulating miRNAs were shown in Additional file 1: Figure S2.

### Model development with the circulating miR-30a-5p/miR-654-5p signature

**Fig. 3:**
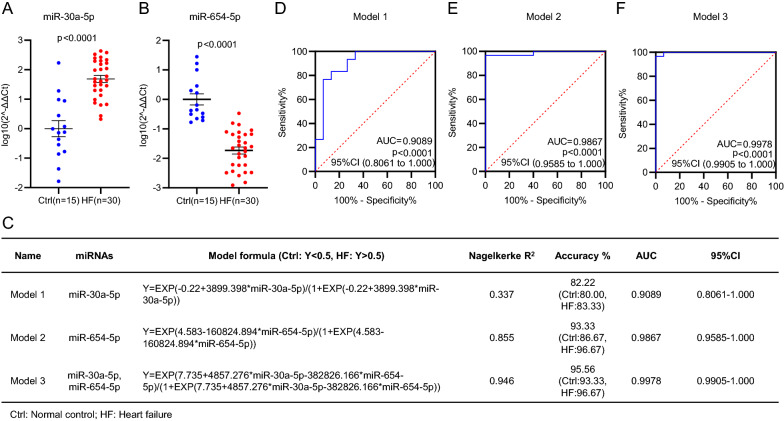
Model development with the circulating miR-30a-5p/miR-654-5p signature in the training cohort. **A**, **B** Quantitative analysis of circulating miR-30a-5p (**A**) and miR-654-5p (**B**) in the 30 HF patients and 15 normal controls (Ctrl) in the training phase. **C** Formulas of the miRNA diagnostic models developed using binary logistic regression method. Three models covering miR-30a-5p (model 1) or miR-654-5p (model 1) and both miRNAs (model 3) were developed and displayed, respectively. **D**–**F** Receiver operating characteristic (ROC) curves of model 1 (**D**), model 2 (**E**) and model 3 (**F**). The values of AUC, 95% CI and p were indicated in the graphs

In order to determine the diagnostic potential of circulating miR-30a-5p and miR-654-5p in HF, the training phase was designed to confirm the expression of circulating miR-30a-5p and miR-654-5p in the plasma samples from 30 HF patients and 15 normal controls. Consistent with the results in the discovery phase, circulating miR-30a-5p showed a significantly higher level while circulating miR-654-5p showed a significantly lower level in HF patients, compared with normal controls (Fig. 3A, B).

To assess the diagnostic values of the two circulating miRNAs, the binary logistic regression method was applied to develop the diagnostic models of the two miRNAs under individual or combination conditions. Three models were derived with the corresponding calculating formulas shown in Fig. 3C, in which Y score > 0.5 was considered as HF, and Y score < 0.5 as normal. In order to assess the specificity and sensitivity of the three models in diagnosis of HF, Receiver Operating Characteristic (ROC) curves were drawn as shown in Fig. 3D–F, in which the Area Under Curve (AUC) was 0.9089, the diagnostic sensitivity was 83.33% and the diagnostic specificity was 80.00% for model 1 (Fig. 3C, D), AUC 0.9867, sensitivity 96.67% and specificity 86.67% for model 2 (Fig. 3C, E), and AUC 0.9978, sensitivity 96.67% and specificity 93.33% for model 3 (Fig. 3C, F). The data suggested more contribution of miR-654-5p to HF diagnosis, and the best diagnostic accuracy of model 3 covering the both miRNAs. These results indicated that even individual miR-30a-5p or miR-654-5p in plasma can be effective in diagnosis of HF, a combination model showed a higher sensitivity and better specificity.

### Independent validation of the circulating miR-30a-5p/miR-654-5p model

**Fig. 4:**
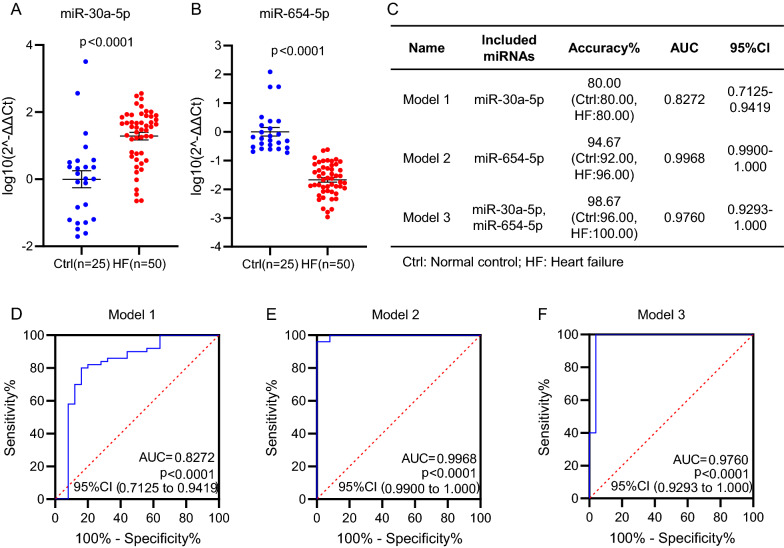
Independent validation of the miRNA models in the validation cohort. **A**, **B** Quantitative analysis of circulating miR-30a-5p (**A**) and miR-654-5p (**B**) in additional 50 HF patients and 25 controls (Ctrl) in the validation phase. **C** Diagnostic accuracy, AUC and 95% CI of the three models tested in the subjects of the validation cohort. **D**–**F** Receiver operating characteristic (ROC) curves of the three models in the validation cohort

In order to further validate the diagnostic values of circulating miR-30a-5p, miR-654-5p and the models developed in the training phase using 30 HF patients and 15 normal controls in Fig. 3, additional 50 HF patients and 25 normal controls were applied in the validation phase for the miRNA analysis and model validation in Fig. 4. Similar to the results in the training phase, here we validated the increased level of circulating miR-30a-5p (Fig. 4A) and the decreased level of circulating miR-654-5p (Fig. 4B) in HF patients, compared with normal controls. The ROC curves also validated the highest sensitivity and the best specificity of model 3 for diagnosis of HF (Fig. 4C–F).

**Fig. 5:**
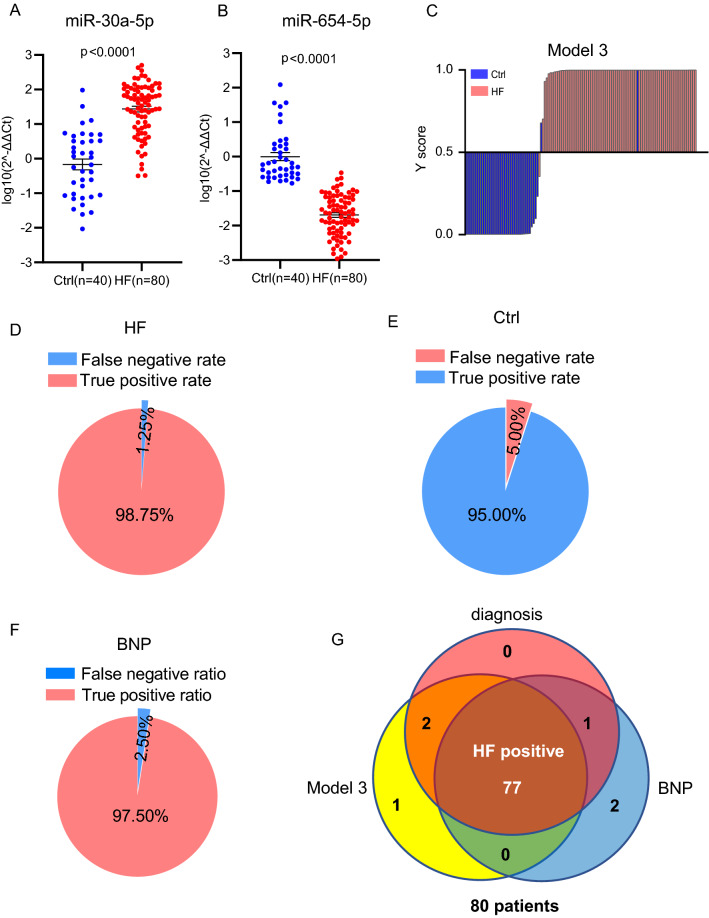
Further validation of the miRNA diagnostic model by combining the two independent clinical cohorts. **A**, **B** Expression levels of circulating miR-30a-5p (**A**) and miR-654-5p (**B**) in the 80 HF patients and 40 controls (Ctrl) combining the training and validation cohorts. **C** Y scores distribution plot of model 3 in the all subjects combining the training and validation cohorts. Y scores greater than 0.5 were judged as HF, otherwise as normal. **D** Pie graph showing the true-positive rate (98.75%) and false negative rate (1.25%) of model 3 in diagnosis of the 80 HF patients. **E** Pie graph showing the true-negative rate (95.00%) and false positive rate (5.00%) of model 3 tested in the 40 normal controls (Ctrl). **F** Pie graph showing the true-positive rate (97.50%) and false negative rate (2.50%) of the 80 HF patients by NT-proBNP-based diagnosis. **G** Venn graph showing overlaps between the clinically diagnosed 80 HF patients, the miRNA model-based model diagnosis and the NT-proBNP-based diagnosis

Next, we combined all subjects in the training phase and validation phase together, including 80 patents and 40 normal controls in total, and applied to confirm the diagnostic value of the models. The abundance levels of circulating miR-30a-5p and miR-654-5p in the 80 patents and 40 controls were shown in Fig. 5A and B. Among the three models, model 3 showed the highest accuracy of 97.50% with AUC 0.9841 as shown in Additional file 1: Figure S3 and S4. When applied for prognosis with the 120 subjects, model 3 gave 98.75% of true positive rate for HF (Fig. 5C, 5D) and 95.00% of true negative rate for normal controls (Fig. 5E). In addition, we compared the prognostic accuracy between the miRNA model we developed and the traditional approach using NT-proBNP level in serum. As shown in Fig. 5F, NT-proBNP-based HF prognosis (following the standard in literature [27]) showed 97.5% of prognostic accuracy (Fig. 5F), a little big lower than our miRNA model (Fig. 5E). Among the 80 clinically diagnosed HF patients, 77 were diagnosed as HF positive by both the miRNA-based model and the NT-proBNP-based approach (Fig. 5G).

The correlations between miR-30a-5p, miR-654-5p and NT-proBNP were further analyzed in the 40 HF patients enrolled in the discovery phase. Comparisons between the levels of miR-30a-5p and miR-654-5p vs the abundance of NT-proBNP in the plasma samples of the HF patients before and after treatment were performed. As shown in Additional file 1: Figure S3, a significantly negative correlation between miR-654-5p and NT-proBNP in plasma of HF patients was observed, which is consistent with the results and the model we developed. As expected, miR-30a-5p showed a positive correlation with NT-proBNP. However, analysis on a larger cohort of HF samples is still required to evaluate its significance.

The correlation between the differential miRNA expression and the NT-proBNP reduction was further analyzed by applying a comparison of the miRNA expression change between 5 patients with ~ 80% NT-proBNP reduction and 5 patients with ~ 40% NT-proBNP reduction upon medical therapy. As shown in the revised Additional file 1: Figure S4, more changes of both miR-654-5p and miR-30a-5p levels were observed in the patients with more NT-proBNP reduction, further suggesting the regulation of the two miRNA levels by medical therapy in HF patients.

**Fig. 6:**
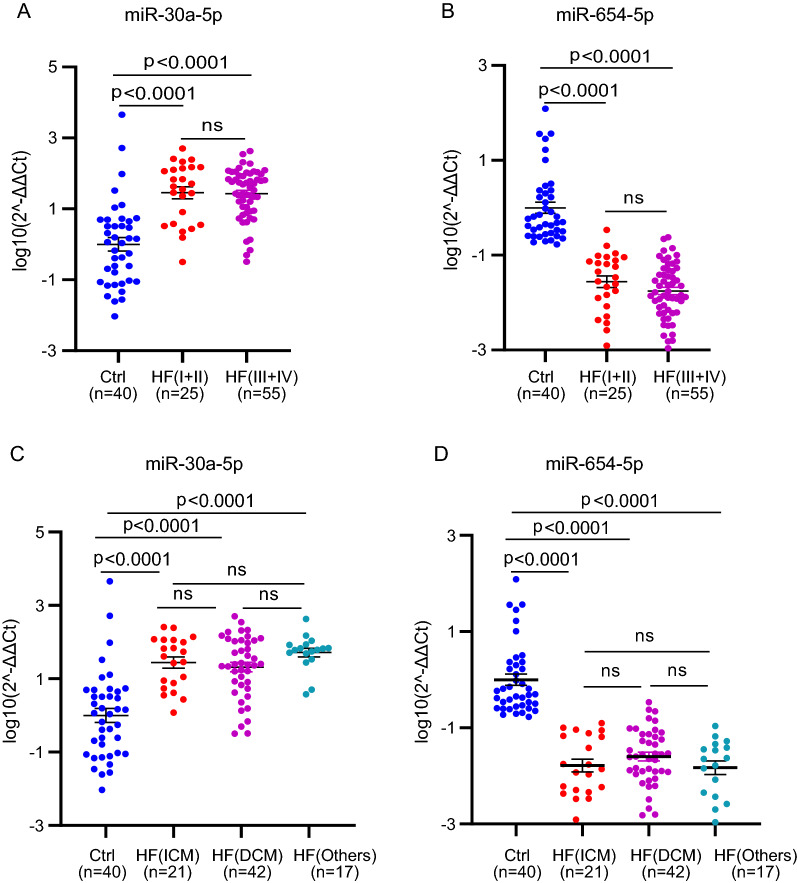
Correlation analysis of the miRNA signature with HF classification and subtypes. **A**, **B** Expression levels of circulating miR-30a-5p (**A**) and miR-654-5p (**B**) in the 40 controls (Ctrl), 25 patients with NYHA class I and II and 55 patients with NYHA class III and IV. **C**, **D** Expression levels of circulating miR-30a-5p (**C**) and miR-654-5p (**D**) in the 40 controls (Ctrl), 21 patients with Ischemic cardiomyopathy (ICM), 42 patients with dilated cardiomyopathy (DCM) and 17 patients with other cardiovascular diseases. p < 0.05 was considered as statistically significance. *ns* means non significant

The correlation between the miR-30a-5p/miR-654-5p signature and pathologic characteristics in the patients was also analyzed. The 80 patients were classified into two groups according to the NYHA Functional Classification, by which 25 patients with NYHA class I and II were grouped to HF(I + II), and 55 patients with NYHA class III and IV were grouped to HF(III + IV). As shown in Fig. 6A and B, the higher level of miR-30a-5p and lower lever of miR-654-5p were indicated in the patients, but did not show significant difference between the two classification groups. Additional analysis between the miRNA signature and cardiomyopathy types in the patients was performed. Cardiomyopathy, as one of the main reasons causing heart failure and even sudden death, is classified to ischemic cardiomyopathy (CM) and dilated non-ischaemic cardiomyopathy (DCM). As seen in Fig. 6C and D, circulating miR-30a-5p showed increase, while circulating miR-654-5p showed decrease in the patients without correlation with the cardiomyopathy types.

### Cardiovascular disease-related target gene analysis of miR-30a-5p and miR-654-5p

**Fig. 7:**
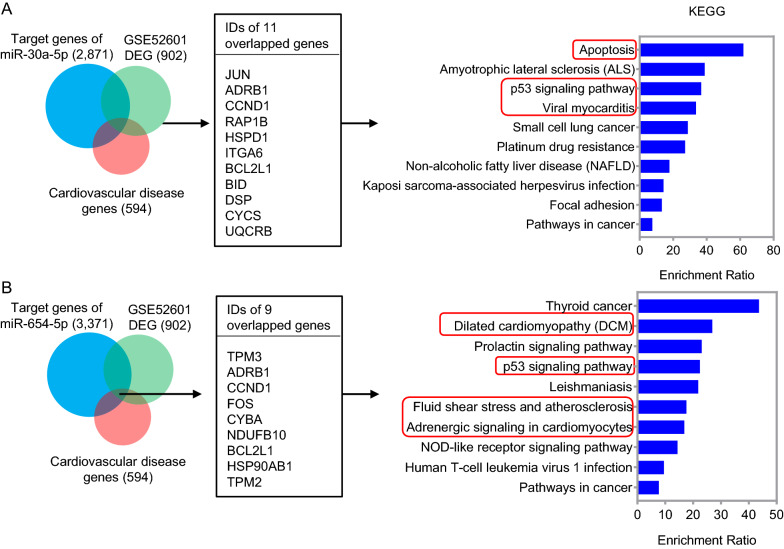
Additional validation of the miRNA signature in association with the therapeutic effect. **A**, **B** Expression levels of circulating miR-30a-5p (**A**) and miR-654-5p (**B**) in 27 HF patients without LVAD implantation (pre_ LVAD), 10 HF patients with LVAD implantation for 3 months (3M_ LVAD) and 10 HF patients with LVAD implantation for 6 months. The miRNA expression data was obtained from dataset GSE53080. p < 0.05 was considered as statistically significance. *ns* means non significant

In order to reveal the function of the two miRNAs, a total of 2871 and 3371 genes were predicted as potential targets of miR-30a-5p and miR-654-5p, respectively, by using ENCORI tool. In addition, 902 differentally expressed genes (DEG) between HF patients and healthy controls were obtained by analyzing a public dataset GSE52601. Among the potential target genes, DEG in HF and cardiovascular disease-related genes in KEGG, 11 genes and 9 genes were overlapped for miR-30a-5p and miR-654-5p, respectively (Additional file 1: Table S4 and S5). KEGG pathway enrichment analysis indicated that miR-30a-5p may involve in pathways regulating apoptosis, p53 signaling, viral myocarditis, et al., and miR-654-5p may regulate pathways of dilated cardiomyopathy (DCM), p53 signaling, atherosclerosis, adrenergic signaling in cardiomyocytes, et al. (Fig. 7).

### Additional validation of the circulating miR-30a-5p/miR-654-5p signature in association with the therapeutic effect

**Fig. 8:**
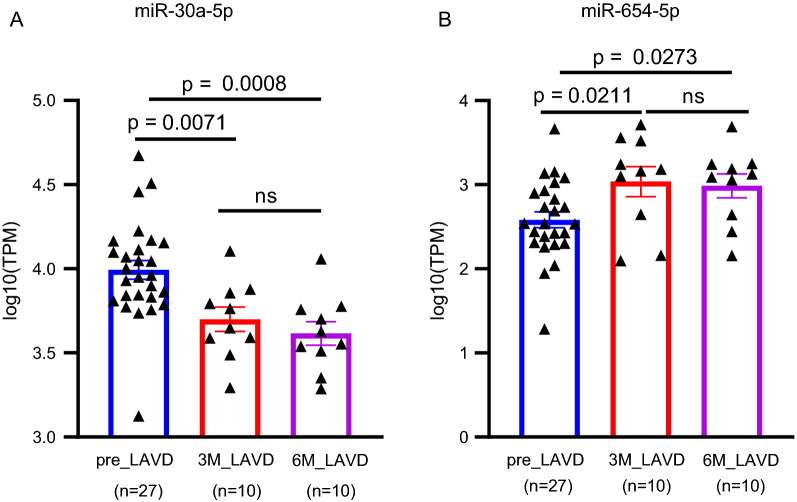
Analysis of the cardiovascular disease-related target genes of miR-30a-5p and miR-654-5p. **A** 11 target genes of miR-30a-5p were overlapped among 2871 predicted target genes, 594 cardiovascular disease genes and 902 DEGs in HF. **B** 9 target genes of miR-654-5p were overlapped among 3371 target genes, 594 cardiovascular disease genes and 902 DEGs in HF. KEGG pathway enrichment analysis was applied by using WebGestalt

In order to further verify the miRNA signature we identified, an external dataset was applied, which contains circulating miRNA profiles in the patients with advanced heart failure with or without medical treatment with left ventricular assist devices (LVAD) implantation. Compared to 27 patients without LVAD implantation, significant decrease of miR-30a-5p (Fig. 8A) and increase of miR-654-5p (Fig. 8B) were found in 10 patients receiving LVAD implantation for 3 months and another 10 patients receiving LVAD implantation for 6 months, which was associated with the improved myocardial function. These data further demonstrated the reliability and utility of the circulating miR-30a-5p and miR-654-5p as biomarkers in diagnosis of HF, and in association with the therapeutic effects as well.

## Discussion

Cardiovascular diseases, as a major public health concern and the leading cause of death and disability globally, are responsible for around 17.18 million deaths every year, representing over 30% of all global deaths according to the health report of World Health Organization in 2019. Most cardiovascular diseases eventually progress to HF, which is associated with a 5-year survival as low as 25% [28]. To make matters worse, the incidence is continuing to increase along with the aging of the general population all over the world. Early diagnosis of cardiovascular diseases has been considered as one of the most promising attempts for reducing the risk and mortality. Identification of new diagnostic biomarkers and development of effective diagnostic models are often the main focus of concern, with potential to be adopted for clinical identification of individuals at high risk for the development of HF.

The present study was started with a comparative analysis of paired plasma samples from same inpatient at the time of check-in before medical treatment and the time of check-out after getting better, which minimized the interference from individual variation of subjects. Six circulating miRNAs were identified in association with medical treatment in HF patients. Among them, the abundances of miR-30a-5p, miR-100, miR-499b, miR-320a and miR-433-3p in plasma showed positive associations, while miR-654-5p showed a negative association with the severity of the heart illness in patients.

Consistent with literature, 5 of the 6 miRNAs we identified have been previously reported to have aberrant expression levels in the circulation and/or heart tissues of patients with cardiovascular diseases. For example, the level of miR-499 increased in the circulation of patients with AMI [16, 17]; circulating miR-30a-5p showed a higher level in the patients with non-ischaemic HF [21]; the expression of miR-100 increased in the tissue samples from both idiopathic and ischemic cardiomyopathies hearts [29]; the levels of circulating miR-320 and miR-433-3p increased in the patients with coronary artery disease [30, 31] and critical coronary stenosis [32], respectively. Consistence with literature is indicative of the reliability of our analysis. Nevertheless, we are the first to identify circulating miR-654-5p as a potential biomarker in the patients with HF. Another novelty is that the current study demonstrated an association of the circulating miRNA levels with the therapeutic effect in HF patients, which provides a potential biomarker for determining the prognosis.

We further confirmed miR-30a-5p and miR-654-5p having a high potential to serve as biomarkers for diagnosis of HF. Accordingly a diagnostic model containing miR-30a-5p and miR-654-5p was developed. The high sensitivity and high specificity of the model were validated through two independent cohorts and an external public dataset, in which the two circulating miRNAs showed consistent correlations with HF. miR-30a-5p has been reported to be highly expressed in the damaged heart [33] and patients with HF, left ventricular dysfunction, and left ventricular hypertrophy [8, 20, 34]. Silencing of miR-30a-5p promoted recovery of cardiac injury, and protected cardiomyocytes from the impact of hypoxia/reoxygenation [35]. Studies of miR-654-5p in regulating cancer has been reported [36]. However, the function of miR-654-5p in the heart remains unclear.

There is a limitation of the two-miRNA model we identified that it was not tested against patients with other disease. As the next step, further validation by multiple-center and large-scale investigation using larger patient cohorts will be of great help to strengthen the value of the two-miRNA model for clinical application. In addition, the tissue origin of circulating miRNAs is still an open question in the field. In HF, we do suppose the change of the levels of circulating miR-30a-5p and miR-654-5p is most likely contributed by the heart tissue and/or the responding cells.

## Supplementary Information

**Additional file 1:** **Table S1.** Characteristics of HF patients in the discovery phase (n=40). **Table S2.** Characteristics of HF patients in the training phase (n=30) and validation phase (n=50). **Table S3.** Home made circulating miRNA panel which was used for the miRNA screening analysis in the discovery phase. **Table S4.** The features of 11 overlapped target genes of miR-30a-5p in Heart Failure (ICM or DCM). **Table S5**. The features of 9 overlapped target genes of miR-654-5p between Heart Failure (ICM or DCM). **Figure S1. **Expression patterns of circulating miR-100, miR-320a, miR-433 and miR-499b-3p in each patient in the discovery phase with paired plasma samples collected at times of check-in and check-out. The p-values showed significant change of the four miRNAs (p < 0.05). **Figure S2. **Expression patterns and change trends of the rest of 83 circulating miRNAs in all patients in the discovery phase. The p-values of these miRNAs did not show significant change. **Figure S3**. Correlation analysis between miR-30a-5p, miR-654-5p and NT-proBNP in the 40 HF patients enrolled in the discovery phase before and after treatment. **Figure S4**. Correlation analysis between the differential miRNA expression change and the NT-proBNP reduction by comparing 5 patients with ~80% NT-proBNP reduction and 5 patients with ~40% NT-proBNP reduction upon medical therapy (n=5, *p < 0.05).

## Data Availability

All data and materials related in this research are available for sharing.

## References

[1] Gedela M, Khan M, Jonsson O (2015). Heart failure. S D Med.

[2] Di Palo KE, Barone NJ (2020). Hypertension and heart failure: prevention, targets, and treatment. Heart Fail Clin.

[3] Salah K, Kok WE, Eurlings LW, Bettencourt P, Pimenta JM, Metra M (2014). A novel discharge risk model for patients hospitalised for acute decompensated heart failure incorporating N-terminal pro-B-type natriuretic peptide levels: a European coLlaboration on acute decompeNsated heart failure: ELAN-HF score. Heart.

[4] Ivey KN, Muth A, Arnold J, King FW, Yeh RF, Fish JE (2008). MicroRNA regulation of cell lineages in mouse and human embryonic stem cells. Cell Stem Cell.

[5] Takaya T, Ono K, Kawamura T, Takanabe R, Kaichi S, Morimoto T (2009). MicroRNA-1 and MicroRNA-133 in spontaneous myocardial differentiation of mouse embryonic stem cells. Circ J.

[6] Zhen L, Zhao Q, Lu J, Deng S, Xu Z, Zhang L (2020). miR-301a-PTEN-AKT signaling induces cardiomyocyte proliferation and promotes cardiac repair post-MI. Mol Ther Nucleic Acids.

[7] Andersen GB, Tost J (2020). Circulating miRNAs as biomarker in cancer. Recent Result Cancer Res.

[8] Maciejak A, Kostarska-Srokosz E, Gierlak W, Dluzniewski M, Kuch M, Marchel M (2018). Circulating miR-30a-5p as a prognostic biomarker of left ventricular dysfunction after acute myocardial infarction. Sci Rep.

[9] Xin Y, Yang C, Han Z (2016). Circulating miR-499 as a potential biomarker for acute myocardial infarction. Ann Transl Med.

[10] Xue S, Liu D, Zhu W, Su Z, Zhang L, Zhou C (2019). Circulating MiR-17–5p, MiR-126–5p and MiR-145–3p are novel biomarkers for diagnosis of acute myocardial infarction. Front Physiol.

[11] Wang Y, Chang W, Zhang Y, Zhang L, Ding H, Qi H (2019). Circulating miR-22-5p and miR-122-5p are promising novel biomarkers for diagnosis of acute myocardial infarction. J Cell Physiol.

[12] Sozzi G, Boeri M, Rossi M, Verri C, Suatoni P, Bravi F (2014). Clinical utility of a plasma-based miRNA signature classifier within computed tomography lung cancer screening: a correlative MILD trial study. J Clin Oncol.

[13] Hanna J, Hossain GS, Kocerha J (2019). The potential for microRNA therapeutics and clinical research. Front Genet.

[14] Shah R, Ziegler O, Yeri A, Liu X, Murthy V, Rabideau D (2018). MicroRNAs associated with reverse left ventricular remodeling in humans identify pathways of heart failure progression. Circ Heart Fail.

[15] Akat KM, Moore-McGriff D, Morozov P, Brown M, Gogakos T, Da Correa Rosa J (2014). Comparative RNA-sequencing analysis of myocardial and circulating small RNAs in human heart failure and their utility as biomarkers. Proc Natl Acad Sci USA.

[16] Olivieri F, Antonicelli R, Spazzafumo L, Santini G, Rippo MR, Galeazzi R (2014). Admission levels of circulating miR-499-5p and risk of death in elderly patients after acute non-ST elevation myocardial infarction. Int J Cardiol.

[17] Yao Y, Du J, Cao X, Wang Y, Huang Y, Hu S (2014). Plasma levels of microRNA-499 provide an early indication of perioperative myocardial infarction in coronary artery bypass graft patients. PLoS ONE.

[18] Kuwabara Y, Ono K, Horie T, Nishi H, Nagao K, Kinoshita M (2011). Increased microRNA-1 and microRNA-133a levels in serum of patients with cardiovascular disease indicate myocardial damage. Circ Cardiovasc Genet.

[19] Xue S, Zhu W, Liu D, Su Z, Zhang L, Chang Q (2019). Circulating miR-26a-1, miR-146a and miR-199a-1 are potential candidate biomarkers for acute myocardial infarction. Mol Med.

[20] Ding H, Wang Y, Hu L, Xue S, Wang Y (2020). Combined detection of miR-21-5p, miR-30a-3p, miR-30a-5p, miR-155-5p, miR-216a and miR-217 for screening of early heart failure diseases. Biosci Rep.

[21] De Rosa S, Eposito F, Carella C, Strangio A, Ammirati G, Sabatino J (2018). Transcoronary concentration gradients of circulating microRNAs in heart failure. Eur J Heart Fail.

[22] McDonagh TA, Metra M, Adamo M, Gardner RS, Baumbach A (2022). 2021 ESC guidelines for the diagnosis and treatment of acute and chronic heart failure. Eur J Heart Fail.

[23] Wilcox JE, Fang JC, Margulies KB, Mann DL (2020). Heart failure with recovered left ventricular ejection fraction: JACC scientific expert panel. J Am Coll Cardiol.

[24] Expert consensus of clinical application of NT-proBNP. Chinese Journal of Cardiovascular Research. 2011; 9(6): 401–408.

[25] Livak KJ, Schmittgen TD (2001). Analysis of relative gene expression data using real-time quantitative PCR and the 2(-Delta Delta C(T)) method. Methods.

[26] Heishima K, Meuten T, Yoshida K, Mori T, Thamm DH (2019). Prognostic significance of circulating microRNA-214 and -126 in dogs with appendicular osteosarcoma receiving amputation and chemotherapy. BMC Vet Res.

[27] Januzzi JL, van Kimmenade R, Lainchbury J, Bayes-Genis A, Ordonez-Llanos J, Santalo-Bel M (2006). NT-proBNP testing for diagnosis and short-term prognosis in acute destabilized heart failure: an international pooled analysis of 1256 patients: the International Collaborative of NT-proBNP Study. Eur Heart J.

[28] Stewart S, MacIntyre K, Hole DJ, Capewell S, McMurray JJ (2001). More ‘malignant’ than cancer? Five-year survival following a first admission for heart failure. Eur J Heart Fail.

[29] Sucharov C, Bristow MR, Port JD (2008). miRNA expression in the failing human heart: functional correlates. J Mol Cell Cardiol.

[30] Chen C, Wang Y, Yang S, Li H, Zhao G, Wang F (2015). MiR-320a contributes to atherogenesis by augmenting multiple risk factors and down-regulating SRF. J Cell Mol Med.

[31] Ren XP, Wu J, Wang X, Sartor MA, Jones K, Qian J (2009). MicroRNA-320 is involved in the regulation of cardiac ischemia/reperfusion injury by targeting heat-shock protein 20. Circulation.

[32] Infante T, Forte E, Punzo B, Cademartiri F, Cavaliere C, Soricelli A (2019). Correlation of circulating miR-765, miR-93-5p, and miR-433-3p to obstructive coronary heart disease evaluated by cardiac computed tomography. Am J Cardiol.

[33] Zhang Y, Cai S, Ding X, Lu C, Wu R, Wu H (2021). MicroRNA-30a-5p silencing polarizes macrophages toward M2 phenotype to alleviate cardiac injury following viral myocarditis by targeting SOCS1. Am J Physiol Heart Circ Physiol.

[34] Pan W, Zhong Y, Cheng C, Liu B, Wang L, Li A (2013). MiR-30-regulated autophagy mediates angiotensin II-induced myocardialhypertrophy. PLoS ONE.

[35] Lv XB, Niu QH, Zhang M, Feng L, Feng J (2021). Critical functions of microRNA-30a-5p-E2F3 in cardiomyocyte apoptosis induced by hypoxia/reoxygenation. Kaohsiung J Med Sci.

[36] Lu M, Wang C, Chen W, Mao C, Wang J (2018). miR-654-5p targets GRAP to promote proliferation, metastasis, and chemoresistance of oral squamous cell carcinoma through Ras/MAPK signaling. DNA Cell Biol.

