# Supplementary material for: Diagnostic potential of a circulating miRNA model associated with therapeutic effect in heart failure
Source: J Transl Med. 2022 Jun 11;20:267. doi: 10.1186/s12967-022-03465-w (PMC9188190; doi:10.1186/s12967-022-03465-w)
Supplement: Supplementary file 1 — Additional file 1: Table S1. Characteristics of HF patients in the discovery phase (n=40). Table S2. Characteristics of HF patients in the training phase (n=30) and validation phase (n=50). Table S3. Home made circulating miRNA panel which was used for the miRNA screening analysis in the discovery phase. Table S4. The features of 11 overlapped target genes of miR-30a-5p in Heart Failure (ICM or DCM). Table S5. The features of 9 overlapped target genes of miR-654-5p between Heart Failure (ICM or DCM). Figure S1. Expression patterns of circulating miR-100, miR-320a, miR-433 and miR-499b-3p in each patient in the discovery phase with paired plasma samples collected at times of check-in and check-out. The p-values showed significant change of the four miRNAs (p < 0.05). Figure S2. Expression patterns and change trends of the rest of 83 circulating miRNAs in all patients in the discovery phase. The p-values of these miRNAs did not show significant change. Figure S3. Correlation analysis between miR-30a-5p, miR-654-5p and NT-proBNP in the 40 HF patients enrolled in the discovery phase before and after treatment. Figure S4. Correlation analysis between the differential miRNA expression change and the NT-proBNP reduction by comparing 5 patients with ~80% NT-proBNP reduction and 5 patients with ~40% NT-proBNP reduction upon medical therapy (n=5, *p < 0.05). [file 12967_2022_3465_MOESM1_ESM.pdf]

Additional file 1

Table S1: Characteristics of HF patients in the discovery phase (n=40).

Table S2: Characteristics of HF patients in the training phase (n=30) and validation phase (n=50).

Table S3: Home made circulating miRNA panel which was used for the miRNA screening analysis in the discovery phase.

Table S4: The features of 11 overlapped target genes of miR-30a-5p in Heart Failure (ICM or DCM).

Table S5: The features of 9 overlapped target genes of miR-654-5p between Heart Failure (ICM or DCM).

Figure S1. Expression patterns of circulating miR-100, miR-320a, miR-433 and miR-499b-3p in each patient in the discovery phase with paired plasma samples collected at times of check-in and check-out. The p-values showed significant change of the four miRNAs ($p < 0.05$).

Figure S2. Expression patterns and change trends of the rest of 83 circulating miRNAs in all patients in the discovery phase. The p-values of these miRNAs did not show significant change.

Figure S3. Correlation analysis between miR-30a-5p, miR-654-5p and NT-proBNP in the 40 HF patients enrolled in the discovery phase before and after treatment.

Figure S4. Correlation analysis between the differential miRNA expression change and the NT-proBNP reduction by comparing 5 patients with ~80% NT-proBNP reduction and 5 patients with ~40% NT-proBNP reduction upon medical therapy (n=5, *$p < 0.05$).

Table S1

Table S1: Characteristics of HF patients in the discovery phase

Characteristics	In-hospital	Out-of hospital	p
Age (mean ± SD)	64.40±11.88	64.40±11.88	
Number (n)	40	40	
LVEF (%)	34.05±5.77		
Physical examination			
Heart rate (bpm)	82.62±17.08	74.24±11.71	0.02
Systolic BP (mmHg)	129.90±22.68	112.92±17.48	<0.01
Diastolic BP (mmHg)	76.62±10.93	70.30±8.98	<0.01
Lab tests			
cTnI (ng/mL)	0.05±0.04	0.05±0.03	0.98
CK-MB (ng/mL)	2.91±1.69	1.68±0.83	<0.01
NT-proBNP (pg/mL)	7803.00±8271.15	2775.16±2142.60	<0.01

Table S2

Table S2: Characteristics of HF patients in the training phases

Characteristics	Training Phase	Training Phase
	Heart Failure	Control
Age (mean±SD)	61.99±13.24	61.08±14.44
Number (n)	80	40
LVEF (%)	32.09±5.86	
Physical examination		
Heart rate (bpm)	83.38±17.37	
Systolic BP (mmHg)	121.12±19.61	
Diastolic BP (mmHg)	74.76±11.04	
Lab tests		
cTnI (ng/mL)	0.05±0.04	
CK-MB (ng/mL)	2.95±2.07	
NT-proBNP (pg/mL)	7040.86±7254.41	7118.92±7821.01
NYHA classification		
Class I (n)	0	
Class II (n)	25	
Class III (n)	42	
Class IV (n)	13	
Cardiomyopathy type		
Ischemic cardiomyopathy (n)	21	
Dilated cardiomyopathy (n)	42	
Others (n)	17	

Table S3

Table S3: Home-made circulating miRNA panel

	1	2	3	4	5	6	7	8	9	10	11	12
A	H2O	5s rRNA	H2O	miR-16	let-7a	let-7b	let-7c	let-7e	let-7f	let-7g	let-7i	miR-10a
B	miR-15a	miR-15b	miR-17	miR-18a	miR-18b	miR-19a	miR-19b	miR-20b	miR-21	miR-23a	miR-24	miR-26
C	miR-27a	miR-27b	miR-29a	miR-29b	miR-30a-5p	miR-30d	miR-92	miR-93	miR-99b	miR-100	miR-103a	miR-106a
D	miR-106b	miR-122	miR-125b	miR-126	miR-129	miR-130a	miR-133a	miR-133b	miR-136	miR-140	miR-143	miR-145
E	miR-150	miR-151-5p	miR-155	miR-181a	miR-181b	miR-182	miR-191	miR-195	miR-199a	miR-199a-3p	miR-208a	miR-214
F	miR-221	miR-222	miR-302a	miR-302c	miR-302d	miR-302e	miR-320a	miR-320b	miR-324-5p	miR-329	miR-342	miR-346
G	miR-369-5p	miR-382	miR-423-3p	miR-423-5p	miR-433	miR-484	miR-486	miR-493	miR-495	miR-499a	miR-499b-3p	miR-499b-5p
H	miR-543	miR-622	miR-638	miR-654	miR-665	miR-675	miR-762	miR-885-5p	miR-16	H2O	5s rRNA	H2O

Table S4

Table S4: The expression features of 11 overlapped target genes of miR-30a-5p

Gene	log2FC ICM Heart Failure - Non-Failing Postnatal	log2FC DCM Heart Failure - Non-Failing Postnatal	t-statistic ICM Heart Failure - Non-Failing Postnatal	t-statistic DCM Heart Failure - Non-Failing Postnatal	P Value ICM Heart Failure - Non-Failing Postnatal	P Value DCM Heart Failure - Non-Failing Postnatal	Ensembl GeneID
JUN	0.9400	0.7930	3.56	2.97	1.92E-03	7.40E-03	ENSG00000177606
HSPD1	0.3260	0.3710	3.20	3.61	4.39E-03	1.71E-03	ENSG00000144381
RAP1B	0.1410	0.1490	2.41	2.52	2.55E-02	2.00E-02	ENSG00000127314
ITGA6	-0.1920	-0.2220	-2.63	-3.01	1.59E-02	6.86E-03	ENSG00000091409
UQCRB	-0.2250	-0.3140	-2.62	-3.62	1.62E-02	1.66E-03	ENSG00000156467
BID	-0.3540	-0.3250	-4.39	-3.99	2.70E-04	7.00E-04	ENSG00000015475
CYCS	-0.3770	-0.3480	-3.51	-3.22	2.13E-03	4.20E-03	ENSG00000172115
DSP	-0.5520	-0.5600	-3.04	-3.05	6.39E-03	6.18E-03	ENSG00000096696
BCL2L1	-0.3930	-0.3800	-3.32	-3.19	3.32E-03	4.55E-03	ENSG00000171552
ADRB1	-0.3660	-0.5650	-2.61	-4.00	1.65E-02	6.90E-04	ENSG00000043591
CCND1	0.5910	0.6250	2.95	3.09	7.88E-03	5.71E-03	ENSG00000110092

Table S5

Table S5: The expression features of 9 overlapped target genes of miR-654-5p

Gene	log2FC ICM Heart Failure - Non-Failing Postnatal	log2FC DCM Heart Failure - Non-Failing Postnatal	t-statistic ICM Heart Failure - Non-Failing Postnatal	t-statistic DCM Heart Failure - Non-Failing Postnatal	P Value ICM Heart Failure - Non-Failing Postnatal	P Value DCM Heart Failure - Non-Failing Postnatal	Ensembl GeneID
BCL2L1	-0.3930	-0.3800	-3.32	-3.19	3.32E-03	4.55E-03	ENSG00000171552
ADRB1	-0.3660	-0.5650	-2.61	-4.00	1.65E-02	6.90E-04	ENSG00000043591
CCND1	0.5910	0.6250	2.95	3.09	7.88E-03	5.71E-03	ENSG00000110092
FOS	2.7900	1.8070	3.47	2.22	2.39E-03	3.77E-02	ENSG00000170345
TPM3	1.3260	1.2130	2.95	2.67	7.79E-03	1.44E-02	ENSG00000143549
HSP90AB1	0.3350	0.3790	3.04	3.41	6.38E-03	2.75E-03	ENSG00000096384
CYBA	-0.2950	-0.2620	-2.46	-2.17	2.29E-02	4.24E-02	ENSG00000051523
NDUFB10	-0.3770	-0.2410	-3.67	-2.32	1.47E-03	3.06E-02	ENSG00000140990
TPM2	-0.5420	-0.6870	-2.12	-2.66	4.65E-02	1.49E-02	ENSG00000198467

Figure S1

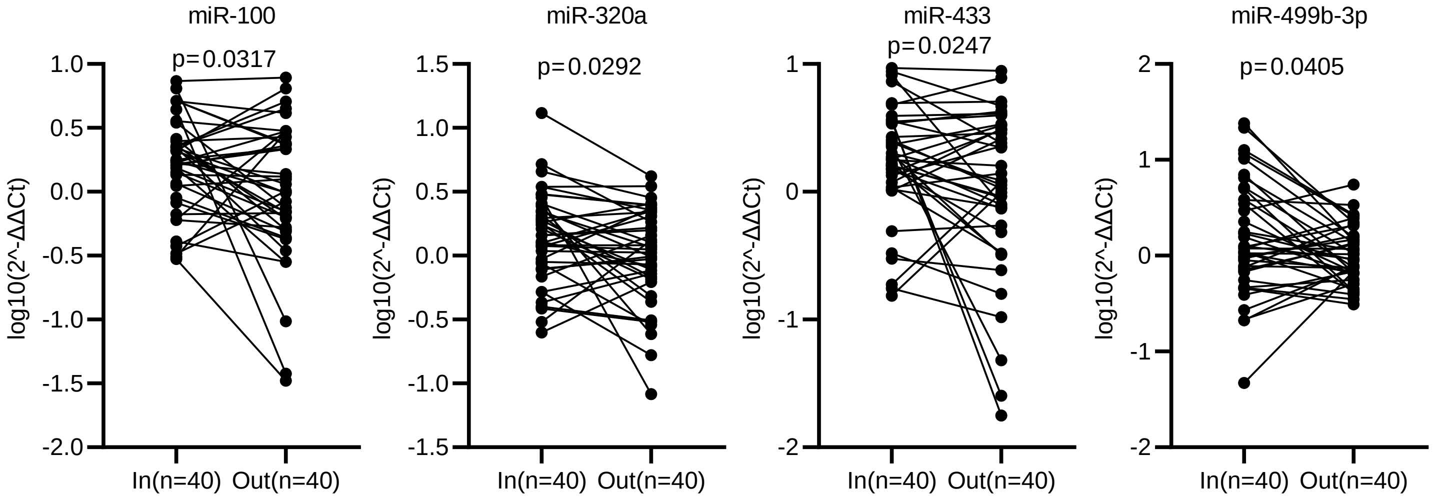
miR-100
p=0.0317
log10(2^-ΔΔCt)
1.0
0.5
0.0
-0.5
-1.0
-1.5
-2.0
In(n=40)
Out(n=40)
miR-320a
p=0.0292
log10(2^-ΔΔCt)
1.5
1.0
0.5
0.0
-0.5
-1.0
-1.5
In(n=40)
Out(n=40)
miR-433
p=0.0247
log10(2^-ΔΔCt)
1
0
-1
-2
In(n=40)
Out(n=40)
miR-499b-3p
p=0.0405
log10(2^-ΔΔCt)
2
1
0
-1
-2
In(n=40)
Out(n=40)

Figure S2

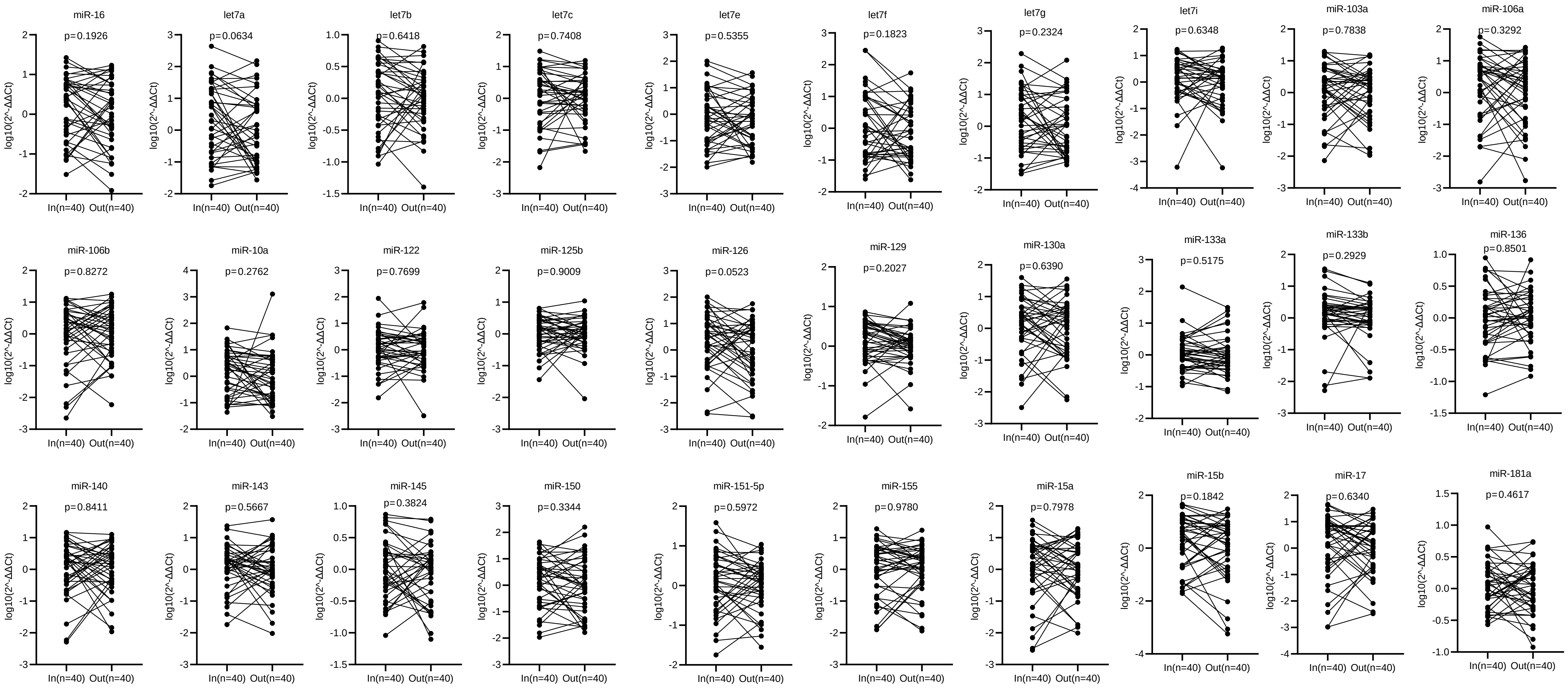

miR-16
p=0.1926
let7a
p=0.0634
let7b
p=0.6418
let7c
p=0.7408
let7e
p=0.5355
let7f
p=0.1823
let7g
p=0.2324
let7i
p=0.6348
miR-103a
p=0.7838
miR-106a
p=0.3292
miR-106b
p=0.8272
miR-10a
p=0.2762
miR-122
p=0.7699
miR-125b
p=0.9009
miR-126
p=0.0523
miR-129
p=0.2027
miR-130a
p=0.6390
miR-133a
p=0.5175
miR-133b
p=0.2929
miR-136
p=0.8501
miR-140
p=0.8411
miR-143
p=0.5667
miR-145
p=0.3824
miR-150
p=0.3344
miR-151-5p
p=0.5972
miR-155
p=0.9780
miR-15a
p=0.7978
miR-15b
p=0.1842
miR-17
p=0.6340
miR-181a
p=0.4617
log10(2^-ΔΔCt)
In(n=40)
Out(n=40)

S2-continued

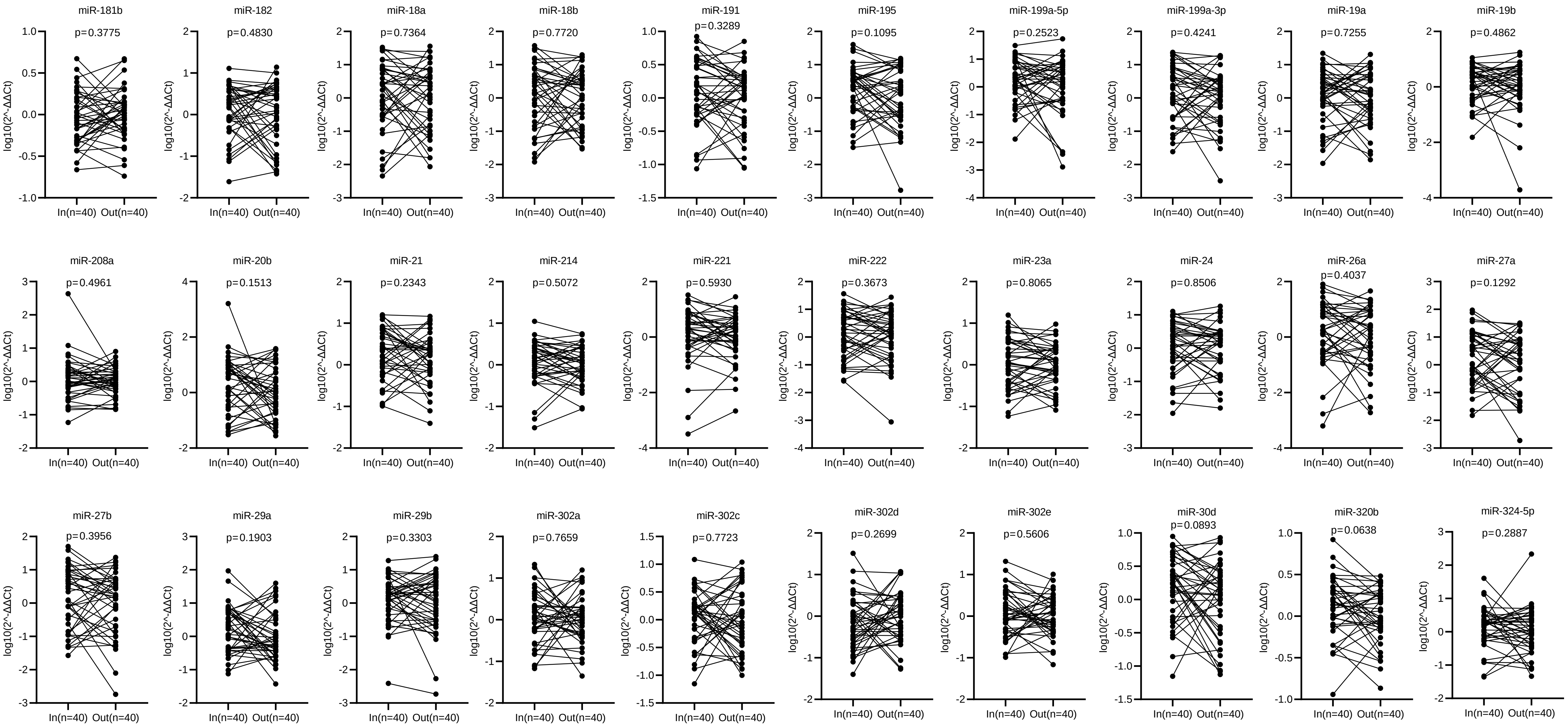

Figure S2-continued

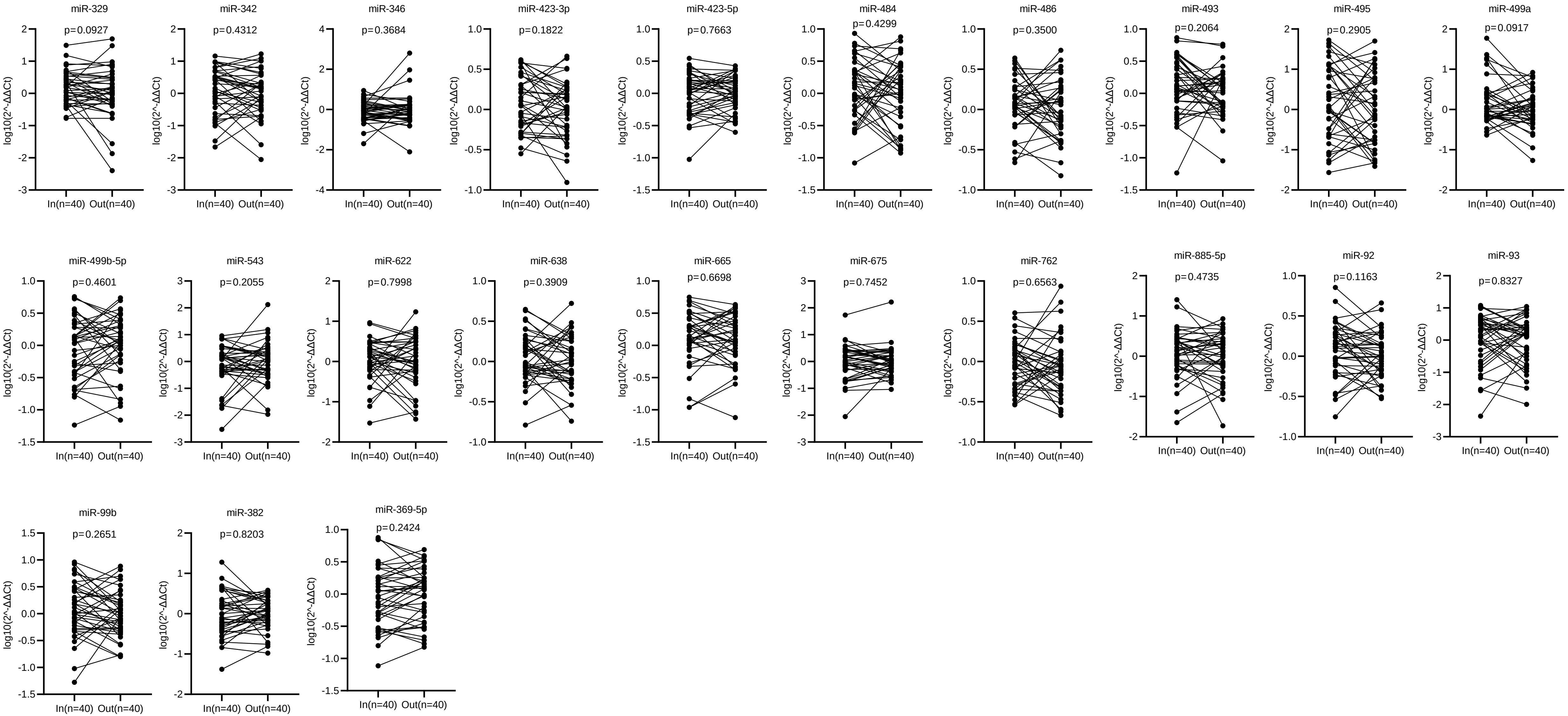

Figure S3

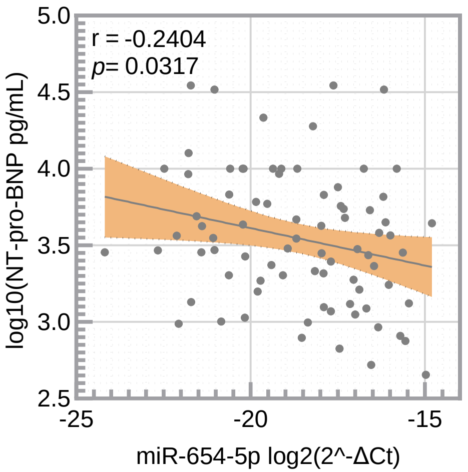

r = -0.2404
p= 0.0317
5.0
4.5
4.0
3.5
3.0
2.5
log10(NT-pro-BNP pg/mL)
-25
-20
-15
miR-654-5p log2(2^-ΔCt)

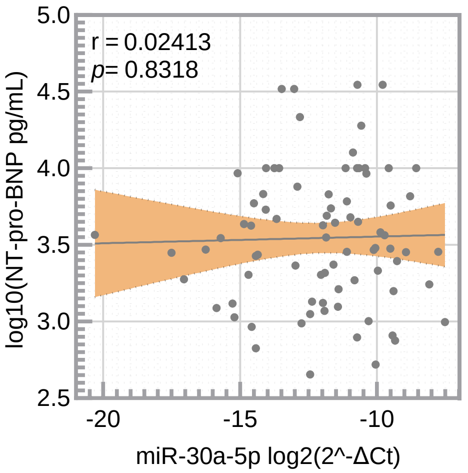

r = 0.02413
p= 0.8318
5.0
4.5
4.0
3.5
3.0
2.5
log10(NT-pro-BNP pg/mL)
-20
-15
-10
miR-30a-5p log2(2^-ΔCt)

Figure S4

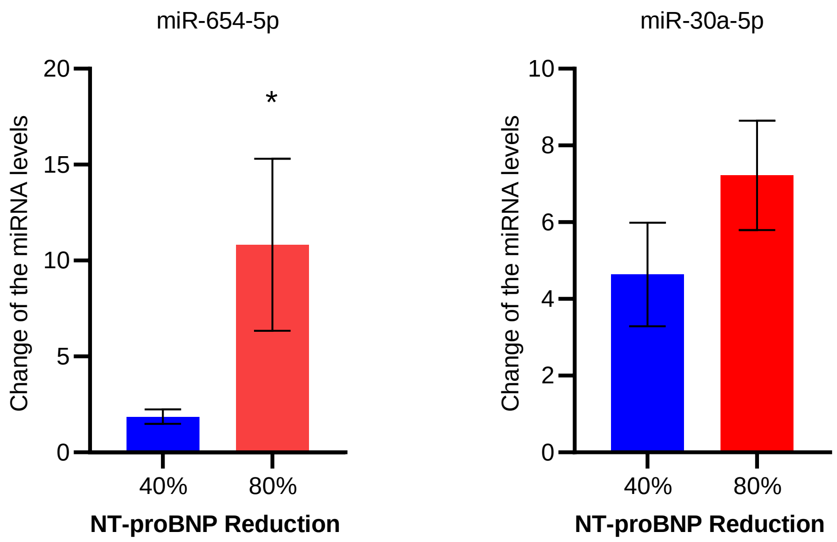
miR-654-5p
Change of the miRNA levels
0
5
10
15
20
*
40%
80%
NT-proBNP Reduction
miR-30a-5p
Change of the miRNA levels
0
2
4
6
8
10
40%
80%
NT-proBNP Reduction
